# Effect of intrauterine bodily development and nutritional status on the later body-length development of children. The MDN system

**DOI:** 10.1186/1687-9856-2013-S1-P206

**Published:** 2013-10-03

**Authors:** Péter Berkő, Kálmán Joubert, Gyula Gyenis

**Affiliations:** 1 Faculty of Health Care, Miskolc University, Borsod-A.-Z. County and University Teaching Hospital, Budapest, Hungary; 2 Demographic Research Institute, Central Statistic Office, Budapest, Hungary; 3 Department of Biological Antropology, Faculty of Science Eötvös Lorand University, Budapest, Hungary

## 

The author’s aim was to study 1./ the effect of intrauterine weight and length development and nutritional status on the later height development of children, - 2./ how can we use the MDN system to identify and distinguish neonates who are likely to need growth hormone treatment in the future.

The authors examined the height of 6335 Hungarian 18 years old young men, whose intrauterine weight and length development was known after birth. They have used a new diagnostic method, so called MDN (Maturity, Development and Nutritional status) system which is suitable to determine the body development and nutritional status of a neonate on the basis of its gestational age, length and weight development considered simultaneously (*Berkő P.*, *Joubert K*. J Maternal Fetal Neonatal Med, 2009; 22/7, pp.552).

Relying on the birth data and the MDN matrix position of 6335 young men, the authors have established, the height of the 18 years old men became smallest who were proportionally retarded neonates at birth time. Their average height was 170.8cm comparing to the young men who were absolutely averages at birth time (176.1). The difference is strongly significant.

The MDN system is a suitable method for the differentiation the mostly endangered neonate groups, based on their body development and nourishment. The development and the nutritional status have a major impact on the neonatal mortality. The MDN system has another important area of application. It allows the prompt and accurate identification of those newborns for whom systematic follow-up measurements and growth hormone therapy treatment is likely to be necessary in the future.

